# Prevalence and associated factors of healthy aging among community-dwelling older adults in Lishui city, China: a cross-sectional study

**DOI:** 10.1186/s12889-025-21420-4

**Published:** 2025-01-16

**Authors:** Jian-Hua Chen, Norhasmah Mohd Zain, Azlina Yusuf, Bi-He Ying

**Affiliations:** 1 School of Health Science, Universiti Sains Malaysia, Health Campus, Kelantan, 16150 Malaysia; 2 Department of Nursing, School of Medicine, Lishui University, No. 1 Xueyuan Road, Lishui City, Zhejiang Province 323000 China

**Keywords:** Depression, Family support, Healthy aging, Health self-management ability, Older adults, Self-perceived economic independence, Self-rated health, Social engagement

## Abstract

**Background:**

Identifying the level of healthy aging and exploring its associated factors are prerequisites in the planning of effective measures among the elderly population. Thus, this study aimed to investigate the prevalence of healthy aging and determine its associated factors among community-dwelling older adults from mountain areas in Lishui, China.

**Methods:**

A multicenter cross-sectional survey was conducted. Participants were recruited by a multi-stage stratified cluster-sampling procedure from a mountainous region in Lishui City, Zhejiang Province, China. A validated questionnaire of Healthy Aging Instruments (HAI), Basic Psychological Needs Satisfaction (BPNS), Patient Health Questionnaire (PHQ-9), Ascertain Dementia 8 questionnaire (AD8), Family Adaption Scale (FAS), Community-Based Health Promotion Activity Questionnaires (HPAQ), Social Function Questionnaire for Chinese Older Adults (SFQCOA), Adult Health Self-Management Skills (ability) Rating Scale (AHSMSRS) was incorporated. The questionnaire also captured sociodemographic characteristics, lifestyle behaviors, and Self-Perceived Healthy Ageing (SPHA). Multivariate stepwise linear regression analysis was performed.

**Results:**

The mean score of the Healthy Aging index was 136.5 (18.22). The majority of the participants have a high level of healthy aging (65.5%). Regression analysis showed 12 predictors of healthy aging: self-perceived economic independence, lifestyle-related behaviors, subjective physical health, psychological health, better competence of BPNS, frequency of community-based HPA participation, lower HPA-perceived barriers, social support, social engagement, and Health Self-Management (HSM) ability with two dimensions HSM-Consciousness and HSM-Behavior, as well as SPHA (*P <* 0.05).

**Conclusions:**

This study contributed to the existing gap in both subjective and objective understanding of healthy aging, especially in terms of its relationship with sociodemographic factors, lifestyle-related behaviors, individual health conditions, environmental coverage, as well as family, community and social support. Both individual-environment interaction factors as better HSM ability as well as SPHA might help predict older adults’ healthy aging in mountainous areas in China. Developing an accurate, reliable health promotion program that provides insights may improve healthy aging changes in mountainous regions for community healthcare staff, especially nurses.

## Introduction

Population aging is a common phenomenon that occurs in many countries around the world. Aging populations are increasingly common in low- and middle-income countries (LMICs). By 2050, two-thirds of the world population above 60 - years of age is expected to be from LMICs [1]. According to a report by WHO, China, an upper middle-income country, the rate of population aging is accelerating at a greater rate in China than in many high-income countries [2]. According to projection [3], China will be a super-aged society (i.e. population aged 65 years and above reaching 20% of the overall population) by 2040. More importantly, in China will likely be highly variable across urban and rural geographical locations. For instance, it is observed in rural areas in China due to the rural-to-urban labor migration in the last few decades. The majority of older people live in rural China. Until 2020, individuals aged 60 - years old and older in rural areas constituted 23.8% of the total population, which is 7.9% higher than their counterparts in urban areas [4]. Mountainous are experiencing a more rapid transition to an aging society compared to cities. Moreover, many of China’s mountainous regions are home to the poorest, least educated, and least healthy population, contrasting sharply with trends observed in high-income countries.

Similarly in the province of Zhejiang, people over 60 - years of age account for 18.7% of the whole province’s population. Lishui, one of the mountainous areas in Zhejiang, recorded an even higher proportion of the aging population (21.2%) in 2020 [5–7]. In other words, Lishui is facing a major problem of rapid aging. In addition, modernization and urbanization have resulted in rural-to-urban labor migration [8]. As a result, there is a steady rise in the number of left-behind older people in rural or mountain areas, translating into a higher dependency ratio, which specifically assesses the ratio of individuals typically not in the labor force (dependents with Individuals aged 0–14 as children and those aged 65 and older as elderly) to those who are (the productive population aged 15 to 64). -This ratio is crucial for understanding the economic burden on the working population and the potential need for social support services [9]. Based on a recent paper, the old-age dependency ratio in rural China is projected to rise sharply up to 79.9% in 2030 and 94.7% in 2050, much higher compared to the urban areas [9]. Furthermore, approximately three in every four older people in China have underlying non-communicable diseases (NCDs) [10]. Compared with their urban counterparts, rural older adults have poorer physical and mental health status [11] with lower healthy aging scores [12]. Therefore, it is vital to determine factors that can improve healthy aging among older adults, especially in rural mountainous areas with resource constraints and logistic challenges.

According to WHO, healthy aging is defined as “a process of developing and maintaining an individual’s functional ability that enables well-being in older age”. Functional ability, in this context, refers to “having the capabilities that enable all people to be and do what they have reasons to value”. This concept encompasses both the intrinsic capacity of the individual (e.g., physical, mental, and cognitive health) and the relevant environmental characteristics (e.g., family, community, and the broader society), as well as the interaction between these elements [13]. Despite the widespread use of the term “healthy aging” in various countries over the past two decades, a precise and universally accepted definition remains elusive. Consequently, there is significant inconsistency in the methods used to operationalize healthy aging across different studies [14].

With regard to the prevalence of healthy aging, previous studies reported a wide range of prevalence among older adults from 3.3 to 53.2% [15, 16]. The disparity is possibly due to the different instruments applied in the studies, thus making it challenging to compare the prevalence of healthy aging between and within countries. In addition, healthy aging is highly multidimensional and complex. According to the original concept of healthy aging, the focus should be placed on functional ability, i.e. the intrinsic capacity of individuals and the interaction with the environment to enable age acceptance, self-sufficiency, simple living, good level of self-care, and self-awareness among older adults. By staying physically and cognitively active, they can continue to have good social participation, social support, and interpersonal relationships, all of which can help to reduce stress and improve well-being [17, 18]. Thus, the healthy aging instrument is necessary to be used to objectively determine the level of healthy aging in the population. Validated instruments are important to guide health care workers (HCWs), especially nurses in performing nursing processes in gerontological practice to promote health and well-being among older adults [19].

In a published systematic review, the concept of healthy aging has been illustrated on a theoretical level whereby the subjective experience of healthy aging was reported based on individuals’ feedback and input, including how they evaluated the spiritual, social, cultural, and psychological dimensions of healthy aging [20]. However, quantitative studies pertaining to an individual’s subjective healthy aging are fewer in the literature. Furthermore, most of the studies focused on older adults from urban or urban-rural areas [12]. To the best of our knowledge, there are no published studies on healthy aging among older adults in mountainous regions, especially in China. Therefore, one of the main objectives of our study was to investigate the prevalence of healthy aging among this marginalized community to address the gap in current research.

As for the second study objective of determining the associated factors of healthy aging, WHO has emphasized the important role played by the combination of individuals’ intrinsic capacity and their surrounding environmental characteristics [2]. A previous review compiled the factors influencing healthy aging across 16 countries and spanning more than half a decade from 1960 to 2022 [20]. The results revealed that individual-environment interaction plays an important role in healthy aging throughout the life course of older adults. Specifically, it highlighted intrinsic factors such as sociodemographic (age, gender, and economic status), health (health, psychological, and cognitive), extrinsic environmental factors (family, community participation, and social function), as well as individual-environmental interaction factors such as healthy self-management ability [21].

The global epidemic of NCDs has not spared the aging population. It is critical to equip the elderly with the necessary health self-management (HSM) ability. They should take the initiative in monitoring and managing their physical functions via biological, psychological, and sociological techniques [22]. This is important because health status is closely related to the quality of life [23], one of the most important elements in healthy aging [24]. While it is generally acknowledged that HSM ability is a major influencing factor of healthy aging, there is a paucity of research that explores the causal relationships between HSM ability and healthy aging [25]. In addition, very few studies are available on the association between individual characteristics such as sociodemographic, lifestyle, health, and environmental conditions with healthy aging. It is particularly vital to explore the interaction between individual-environmental factors and healthy aging. Given the rapid aging transition in China and the existing gaps in the local research on the aging population, there is an urgent need to comprehensively explore healthy aging among older adults, especially in mountainous areas of China. Therefore, the second objective of this study aimed to determine the associated factors of healthy aging among community-dwelling older adults from Lishui, China to establish the necessary evidence that can guide the development of effective interventions.

## Materials and methods

### Research design & setting

The cross-sectional design was conducted among community-dwelling older adults from Liandu district within Lishui City, Zhejiang Province, China. Due to the high proportion of the aging population in Lishui City, it came across as a suitable study site to reflect the healthy aging issue in the province and country as a whole. Additionally, Liandu district serves as the administrative, political, economic and cultural center in Lishui and comprises six sub-districts. For this study, six sub-districts were chosen through a non-random selection process. Subsequently, communities within each subdistrict were selected using random procedures. Finally, elderly individuals residing in community dwellings were identified through random cluster sampling.

### Research population

The participants in this study were recruited using a multistage, stratified cluster sampling procedure. The sample size calculation was based on the prevalence of healthy aging from a previous study (28.2%) [15, 16].

Based on the rate sample size formula method [26]: $n = \left({{{{{{Z_{1 - \alpha /2}}} \over \delta }}^2}\,p(1 - p)} \right)$

With δ = allowable error (generally half of the confidence interval), ${Z_{1 - \alpha /2}}$= coefficient of standard deviation (usually expressed as 1.96 for 95% confidence interval), a total of 374 subjects was needed for this study. The sample size was further inflated by 20% to account for possible missing data, giving rise to a total sample size of 390. The inclusion criteria were (a) resided in the local community within Liandu district of Lishui City for at least one year; (b) 60 years old or above; (c) able to communicate in Mandarin Chinese and the local language; and (d) understand and able to respond to the questions in the questionnaire. The exclusion criteria were (a) history of chronic alcoholism or brain trauma; (b) hearing impairment; (c) speech impairment; (d) cognitive impairment and inability to communicate verbally; (e) severe mental illness; (f) severe or terminal illness; and (g) those who lived in nursing homes.

#### Instrument

The study instrument consisted of a self-developed questionnaire and a standard questionnaire. The self-developed questionnaire included sociodemographic characteristics, lifestyle-related behaviors, and health-related variables. The standard questionnaires included 35 items from Healthy Aging Instruments (HAI), Basic Psychological Needs Satisfaction (BPNS), Patient Health Questionnaire (PHQ-9), Ascertain Dementia 8 questionnaire (AD8), Family Adaption Scale (FAS), Community-Based Health Promotion Activity Questionnaires (HPAQ), Social Function Questionnaire for Chinese Older Adults (SFQCOA), Adult Health Self-Management Skills (ability) Rating Scale (AHSMSRS) as detailed in the following section:

**1) Healthy Aging Instruments (HAI)** was originally developed in Thailand to measure the healthy aging level among community-dwelling older adults [17]. Subsequently, it was adapted to be used in the Chinese context [18].It comprises a total of 35 items under nine dimensions: (1) Being self-sufficient and living simply, (2) Managing stress, (3) Having social relationships and support, (4) Making merit and good deeds, (5) Practicing self-care and self-awareness, (6) Staying physically active, (7) Staying cognitively active, (8) Having social participation, and (9) Ageing acceptance.

Each item in the instrument is rated on a five-point Likert scale (1 = completely inconsistent, 2 = slightly consistent, 3 = uncertain, 4 = largely consistent, and 5 = fully consistent). The total score ranges between 35 and 175 points, with a higher score indicating a higher level of HA. The total score was categorized into: low level (35.00-81.66), medium level (81.67-128.33), and high level (128.34–175.00) [27]. The Cronbach’s alpha value for the overall instrument was 0.88, ranging from 0.69 to 0.80 for each dimension [17]. In addition, the Chinese version of HAI also has good reliability and validity with a Cronbach’s alpha value of 0.92 [18]. Additionally, Self-Perceived Healthy Aging (SPHA) was also incorporated to evaluate the subjective experience of HA because of the multidimensionality of HA [20]. SPHA can be ranged from poor to very good on a five-point Likert scale (1 = poor, 2 = less poor, 3 = general, 4 = good, 5 = very good).

**2) Sociodemographic characteristics** included age (60 years old and above), gender (male vs. female), Nationality (Han vs. Non-Han), residence (urban vs. non-urban), religion (none vs. yes) educational background (primary school and below vs. junior school and above), past occupation (farmer, workers and other), marital status (married vs. other), living arrangements (Living alone vs. living with other), personal income monthly (less than 3001RMB vs. 3001 and above RMB), self-perceived economic independence (dependency vs. independence), fix the source of income (no vs. yes), and medical insurance (no vs. yes).

**3) Lifestyle-related behaviors** were assessed using five questionnaires, including sleep hours (less than 6 h vs. 6 h and above), smoking (no vs. yes), alcohol consumption (no vs. yes), physical activity (no vs. yes), and smartphone use (no vs. yes).

**4) Health-related variables** were evaluated from three dimensions, i.e. physical-mental-cognitive health states based on a WHO report on the concept of health [2].

**4a) Physical health conditions** were evaluated using three self-reported questions, i.e. chronic diseases (yes vs. no), number of falls in the past year (yes vs. no), and Self-Rated Health (SRH). The self-reported chronic disease was verified by checking the medical diagnosis with a registered medical practitioner. SRH was determined by asking “How do you rate your health?” on a scale of (1) poor (1) to (5) very good.

**4b) Mental health conditions** were assessed by four main dimensions that consisted of depression, loneliness, Basic Psychological Needs Satisfaction (BPNS), and self-perceived happiness. Depression was evaluated by the Patient Health Questionnaire (PHQ-9) [28], a nine-item depression module to screen for common mental disorders. Each item is scored from 0 to 3 (0 = not at all, 1 = several days, 2 = more than half the days, and 3 = nearly every day), giving a total score ranging from 0 to 27. A higher score indicates a more severe level of depression. PHQ-9 (Chinese version) is a reliable and valid measure for assessing depression among community-dwelling older adults with a Cronbach’s alpha value of 0.832 [29]. Loneliness was determined via Self-perceived loneliness (SPL), i.e. “Do you suffer from loneliness?” Participants were categorized according to their responses on a five-point Likert scale (1 = never, 2 = seldom, 3 = sometimes, 4 = often, and 5 = always). Next, the BPNS scale which comprises nine items under three subscales (autonomy, competence, and relatedness) was also included [30]. Each item can be scored on a seven-point Likert scale from 1 to 7 (1 = strongly disagree, 2 = disagree, 3 = relatively disagree, 4 = average, 5 = relatively agree, 6 = agree, 7 = strongly agree). The average score of three items in each dimension reflects the need satisfaction of that dimension while the average score of all nine items reflects the satisfaction of the total needs with a higher score indicating higher satisfaction. The total Cronbach’s alpha value of the scale was 0.877, with 0.826 (autonomy), 0.807 (competence), and 0.847 (relatedness) for each subscale. The instrument has been translated into the Chinese language and has shown good reliability and validity among the elderly [30]. Lastly, Self-perceived Happiness (SPH) was evaluated by asking: “Overall, do you feel happy in your current life?” on a seven-point Likert scale (1 = very unhappy, 2 = unhappy, 3 = relatively unhappy, 4 = generally, 5 = relatively happy, 6 = happy, and 7 = very happy).

**4c) Cognitive function** was assessed using the Ascertain Dementia 8 questionnaire (AD8) [31], a self-rating tool to determine an individual’s cognitive status. It can differentiate between signs of normal aging and mild dementia. It comprises eight items to be answered as Yes (1) or No (0) [32]. The sum of the eight items ranged from 0 to 8 whereby scores of 0 and 1 indicate normal cognition while any scores above 2 reflect an increasing severity of impairment.

**5) Environmental-related variables** include family conditions, community participation, and social function. According to the WHO, environmental factors are extrinsic factors that can influence healthy aging and they incorporate family, communities, and the broader society that form the context of a person’s life [2].

**5a) Family conditions** were assessed by using the Chinese version of the Family Adaption Scale (FAS) [33]. It contains 15 items on three subscales, (i. family interpersonal relationships, ii. family structure and function, and iii. relationship with the community and natural environment). All items are rated on a seven-point Likert scale, from “a family which is not at all adjusted” (1) to “a completely adjusted family” (7). Higher scores indicate a better level of family adaptation. The Cronbach’s alpha value of the total scale was 0.951, with 0.901 (family interpersonal relationships), 0.885 (family structure and function), and 0.860 (family relationship with community and natural environment) for the three respective subscales [33].

**5b) Community participation** was assessed by Community-Based Health Promotion Activity Questionnaires (HPAQ) [34]. The first part of HPAQ explores the frequency of elderly participation in community-based health promotion activities (1 item) while the second part assesses perceived benefits (19 items), perceived barriers (17 items), self-efficacy (10 items), social support (14 items), and activity-related affect (9 items). All items are rated using a five-point Likert scale ranging from 1 (completely disagree/not confident at all) to 5 (completely agree/very confident). Higher scores indicate better benefits, self-efficacy, social support, and positive effects. All negatively worded items in the questionnaire are reverse-coded. All items reported Cronbach’s alpha scores of above 0.88. The coefficients for each dimension were 0.94 (perceived benefits), 0.91 (perceived barriers), 0.84 (self-efficacy), 0.72 (social support), and 0.94 (activity-related effect) [34]. The questionnaire was demonstrated to have good reliability and validity based on a study conducted in Taiwan.

**5c) Social function** was assessed by the Social Function Questionnaire for Chinese Older Adults (SFQCOA) [35] which consists of 12 items in three dimensions: social support, social adaptation, and social engagement. Each item is rated using a four-point Likert scale (1 = Disagree, 2 = Somewhat disagree, 3 = Somewhat agree, and 4 = Agree). Items of social adaptation are all scored reversely. The Cronbach’s alpha score of the total SFQCOA was 0.75, with 0.74 (social support), 0.77 (social adaptation), and 0.82 (social engagement) for each dimension [35].

**6) Health self-management ability factors** were assessed by the Adult Health Self-Management Skills (Ability) Rating Scale (AHSMSRS) which was specially designed according to the Chinese cultural context by Chinese researchers [21]. It comprises a total of 38 items across three dimensions, namely health self-management cognition (HSMC) (14 items), health self-management behavior (HSMB) (14 items), and health self-management environment (HSME) (10 items). The total scores range from 38 to 190, with a higher score indicating better health self-management ability. The overall Cronbach’s alpha value is 0.933 [21]. The scale has been demonstrated to have good reliability and validity in assessing health self-management abilities in the elderly [21, 36].

### Data collection

Data collection was conducted from February to April 2023 by a survey team comprising one principal investigator (PI) and ten research assistants. To ensure inter-rater reliability, the PI provided training to the survey team on the questionnaire content and survey techniques. Prior to the data collection, the PI contacted leaders of the selected residential areas in Lishui to obtain permission for the study. Additionally, all older adults in these areas were informed one day in advance about the research team’s visit. The survey team then visited the selected residential communities to identify potential participants. Individuals were screened for their eligibility before being briefed on the study’s purpose and procedures. All participants were informed that participation was voluntary and anonymous, and they were assured that confidentiality would be maintained. After obtaining written consent, participants completed a self-administered questionnaire. For those with low literacy levels or poor vision, research assistants provided assistance by reading the questions aloud and recording participant’s responses. The completed questionnaires were subsequently reviewed and collected by the surveyors.

### Data analysis

Epidata software version 3.2 was employed for data entry and SPSS 26.0 (US SPSS Inc.) was utilized for data analysis. Descriptive statistics were applied to summarize the social-demographic characteristics. The Kolmogorov–Smirnov test, along with a histogram normal curve, was used to assess the normal distribution of the HAI scores. The results of the normality test indicated that the histogram curve was symmetrical, suggesting a normal distribution. Therefore, mean (M), standard deviation (SD) and frequencies were calculated to describe the baseline data characteristics. Differences between the categories of healthy aging and the studied variables were analyzed using analysis of variance (ANOVA) for continuous variables and the t-test for categorical variables. Additionally, linear regression analysis was performed with the healthy aging index score as the dependent variable. Predictor variables with *P-value* ≤ 0.05 were included in a stepwise linear regression analysis, while multivariate linear regression was employed to explore the factors influencing healthy aging. A *P-value* < 0.05 was considered statistically significant across all analyses.

## Results

### Participants’ demographic characteristics

A total of 390 community-dwelling older adults participated initially. However, one participant dropped out due to incomplete the questionnaire and only 389 participants completed all the items in the questionnaires (response rate 99.7%). The average age of study participants was 72.1 (SD = 8.34) years, with females accounting for more than half (*n* = 260, 66.8%) of them. Almost all of them had a fixed source of income (*n* = 383, 98.5%) with approximately 66.0% reporting a monthly personal income of less than 3000 RMB (*n* = 255). The medical insurance coverage rate was high (*n* = 380, 97.7%). More than 80.0% of the participants lived with others (*n* = 332, 85.3%) and residence in the city community in Lishui (*n* = 323, 83.0%). More than 70% of them were self-perceived economically independent (*n* = 292, 75.1%), not religious (*n* = 281, 72.2%) and young elderly aged 60 to 74 (*n* = 249, 64.0%). About half (50%) had a low educational level of primary school or below (*n* = 208, 53.5%) and worked as farmers before (*n* = 182, 46.8%) (Table 1).

**Table 1:** Socio‑demographic and healthy aging scores of older adults in Lishui City, China (*n* = 389)

Variables	*n* (%)	Healthy aging scores M (SD)	t/F	*P*-value
Age (years old)			2.878	0.004^a**^
≤ 74	249 (64.0)	138.47 (18.42)		
≥ 75	140 (36.0)	132.99 (17.37)		
Gender			-1.995	0.047^a*^
Male	129 (33.2)	133.89 (18.22)		
Female	260 (66.8)	137.79 (18.12)		
Nationality			0.255	0.799^a^
Han nationality	376 (96.7)	136.54 (18.06)		
Non-Han nationality	13 (3.3)	135.23 (23.30)		
Place of Residence			-2.949	0.003^a**^
Urban area	323(83.0)	137.71 (18.66)		
Non-Urban area	66 (17.0)	130.53 (14.62)		
Religious			0.080	0.936^a^
None	281 (72.2)	136.54 (18.58)		
Yes	108 (27.8)	136.38 (17.33)		
Education			-6.545	< 0.001^a***^
Primary school and below	208 (53.5)	131.14 (17.46)		
Junior high school and above	181 (46.5)	142.66 (17.14)		
Past Occupation			10.022	< 0.001^b***^
Farmer	182 (46.8)	132.53 (17.56)		
Worker	115 (29.6)	141.92 (19.52)		
Others	92 (23.7)	137.58 (15.98)		
Marital status			3.508	0.001^a***^
Married	290 (74.6)	138.37 (18.46)		
Other	99 (25.4)	131.03 (16.41)		
Living arrangements			-2.562	0.011^a*^
Living alone	57 (14.7)	130.82 (17.39)		
Living with others	332 (85.3)	137.47 (18.21)		
Personal income/monthly			-7.328	< 0.001^a***^
≤ 3000 RMB	255 (65.6)	131.89 (17.07)		
≥ 3001 RMB	134 (34.4)	145.26 (17.15)		
Self-perceived economic independence			6.451	< 0.001^a***^
Yes	292 (75.1)	139.77 (17.94)		
No	97 (24.9)	126.66 (15.37)		
Fix the source of income			-2.500	0.013^a*^
No	6 (1.5)	118.17 (15.28)		
Yes	383 (98.5)	136.79 (18.13)		
Medical insurance			-2.677	0.008^a**^
No	9 (2.3)	120.56 (8.08)		
Yes	380 (97.7)	136.88 (18.23)		

Statistical test - ^a^Independent t-test, ^b^ANOVA and multiple comparisons (LSD) were used to compare the groups; ^*^ indicates statistically significant P-values, ^*^P < 0.05, ^**^P < 0.01, ^***^P < 0.001; M = Mean, SD = standard deviations (the same as below in the text)

## Healthy aging score and self-perceived healthy aging

The participants in this study showed a high level of healthy aging with a mean score of 136.5 (18.22) ranging from 90 ~ 175. The highest score among the nine dimensions was the dimension of “accepting aging”, with a mean score of 4.6 (0.59). The lowest score from the nine dimensions was the “social participation dimension”, with a mean score of 2.68 (1.45) (Table 2).

**Table 2:** Healthy aging scores among community-dwelling older adults in Lishui, China (*n* = 389)

Items	M (SD)	Score range	Item average score M (SD)	Minimum	Maximum
Dimension 1 Being Self-Sufficient and Living Simply	21.41 (3.00)	9 ~ 25	4.28 (0.60)	1.00	5.00
Dimension 2 Managing Stress	21.13 (4.44)	5 ~ 25	4.23 (0.89)	1.00	5.00
Dimension 3 Having Social Relationships and Support	17.20 (3.47)	4 ~ 20	4.30 (0.88)	1.00	5.00
Dimension 4 Making Merit and Good Deeds	17.23 (3.16)	4 ~ 20	4.31 (0.79)	1.00	5.00
Dimension 5 Practicing Self-Care and Self-Awareness	16.89 (2.90)	4 ~ 20	4.22 (0.73)	1.00	5.00
Dimension 6 Staying Physically Active	13.40 (4.00)	4 ~ 20	3.35 (1.00)	1.00	5.00
Dimension 7 Staying Cognitively Active	12.05 (4.81)	4 ~ 20	3.01 (1.20)	1.00	5.00
Dimension 8 Having Social Participation	8.05 (4.35)	3 ~ 15	2.68 (1.45)	1.00	5.00
Dimension 9 Accepting Aging	9.13 (1.18)	5 ~ 10	4.57 (0.59)	1.00	5.00
Total score	136.50 (18.22)	90 ~ 175	3.90 (0.52)	35.00	175.00

Notes: Abbreviations: M = Mean, SD = standard deviations

Regarding the prevalence of healthy aging, about 65.5% (*n* = 255) of participants reported a high level of healthy aging (scores 128.34 ~ 175.00) while the remaining 34.4% (*n* = 134) of participants had a medium level (scores 81.67 ~ 128.33) of healthy aging. No participants reported a low level of healthy aging (35.00 ~ 81.66). In terms of self-perceived healthy aging (SPHA), about 79.7% (*N* = 280) of participants reported a good/ very good level of healthy aging while 20.3% (*N* = 79) of them reported a poor level of healthy aging (Table 3). In summary, about two-thirds (65.5%) of participants showed a high level of objective and subjective perception of healthy aging. More importantly, the subjective perception of good healthy aging (79.7%) was higher than their objective perception of high healthy aging (65.5%).

**Table 3:** Self-perceived healthy aging (SPHA) among older adults community-dwelling in Lishui, China (*n* = 389)

Self-perceived healthy aging status	*N* (%)
Less poor	7 (1.8)
General	72 (18.5)
Good	258 (66.3)
Very good	52 (13.4)

Notes: No elderly people self-perceived HA as poor. Dichotomy was noted in the responses: poor = 1, less poor = 2, general = 3, good = 4, and very good = 5 (response alternative from poor, less poor to general as 1–3) and good (response good to very good as 4–5). N = number, (%) = percentage

### Lifestyle-related behaviors

Regarding lifestyle-related behaviors, more than 90% of participants used phones (92.8%). About 67.1% were smartphone users. 83.2% of them had never smoked and 74.3% never consumed alcohol before. In addition, 41.4% of them slept less than six hours per night. More critically, nearly 20% had never participated in any physical activity (Table 4).

**Table 4:** Lifestyle-related variables and healthy aging scores of older adults in Lishui, China (*n* = 389)

Lifestyle-related variables	*N* (%)	Healthy aging scores M(SD)	t	*P*-value
Sleep hours/night			-1.547	0.123
< 6 h	161 (41.4)	134.80 (17.86)		
≥ 6 h	228 (58.6)	137.70 (18.42)		
Smoking				
No	320 (82.3)	137.10 (18.18)	1.411	0.159
Yes	69 (17.7)	133.70 (18.29)		
Drink alcohol			3.003	0.003^**^
No	289 (74.3)	138.11 (18.57)		
Yes	100 (25.7)	131.83 (16.38)		
Participate physical activity			-3.223	0.001^**^
No	84 (21.6)	130.89 (15.40)		
Yes	305 (78.4)	137.09 (19.39)		
Phone use			1.249	0.212
Yes	361 (92.8)	136.82 (17.89)		
No	28 (7.2)	132.36 (22.00)		
Smartphones use			6.631	< 0.001^***^
Yes	261 (67.1)	140.62 (17.24)		
No/Unknown	128 (32.9)	128.24 (17.30)		

Notes: Statistical test - Independent t-test, Significant difference at ^*^P < 0.05, ^**^P < 0.01, ^***^P < 0.001; M = Mean, SD = standard deviations

### Factors associated with healthy aging

#### Individual related factors

The following demographic factors were significantly associated with healthy aging scores (*p* < 0.05), i.e. age, gender, residence, education, past occupation, marital status, living arrangements, personal income, self-perceived economic independence, fixed source of income, and medical insurance coverage (Table 1). In terms of lifestyle behavior factors, alcohol consumption, participation in physical activity, and smartphone usage were significantly associated with healthy aging (*p* < 0.05).

Individual health-related factors that included the physical, mental, and cognitive functions of the participants were presented in Table 5. Based on the simple linear regression, the number of falls in the past 12 months, SRH, depression, loneliness, BPNS, happiness, and cognitive function were significantly associated with healthy aging scores (*p* < 0.05).

**Table 5:** Individual factors, environmental factors, HSM ability, SPHA, and healthy aging scores of older adults in Lishui City, Zhejiang China (*n* = 389)

Factors	Variable	B	95% (CI)	*P*-value^c^	Healthy aging scores M(SD)/*r*^2^
**Individual health-related factors**					
Physical health	Chronic diseases	-2.121	(-5.899 ~ 1.656)	0.270	0.003^a^
	No				137.85 (18.30)^b^
	Yes				135.73 (18.17)^b^
	Fall in the past 12 months	-7.309	(-12.224 ~ -2.394)	0.004	0.022^a^
	No				137.66 (18.07)^b^
	Yes				130.35 (17.93)^b^
	SRH	7.901	(5.882 ~ 9.920)	< 0.001	0.133^c^
Psychological health	Depression	-2.265	(-2.835 ~ -1.694)	< 0.001	0.136^c^
	Loneliness	7.260	(5.541 ~ 8.979)	< 0.001	0.151^c^
	Basic psychological needs satisfaction (BPNS) total score	10.138	(8.499 ~ 11.776)	< 0.001	0.277^c^
	-BPNS-autonomy	5.004	(3.615 ~ 6.393)	< 0.001	0.115^c^
	-BPNS-competence	4.754	(3.474 ~ 6.034)	< 0.001	0.121^c^
	-BPNS-relatedness	7.599	(6.298 ~ 8.901)	< 0.001	0.254^c^
	Happiness	7.345	(5.773 ~ 8.917)	< 0.001	0.179^c^
Cognitive	Cognitive function	-1.845	(-2.667 ~ -1.023)	< 0.001	0.048^c^
**Environmental-related factors**					
Family	Family adaption total scores	9.519	(7.963 ~ 11.075)	< 0.001	0.272^c^
	-Family’s interpersonal relationships	1.640	(1.350 ~ 1.931)	< 0.001	0.242^c^
	-Family’s structure and function	1.872	(1.555 ~ 2.189)	< 0.001	0.256^c^
	-Family’s relationship to the community and the nature environment	2.262	(1.881 ~ 2.643)	< 0.001	0.260^c^
Community	Frequency of elderly participation in community-based health promotion activities	7.729	(6.471 ~ 8.986)	< 0.001	0.274^c^
	HPAQ-Perceived benefits	0.603	(0.512 ~ 0.694)	< 0.001	0.305^c^
	HPAQ-Perceived barriers	-0.643	(-0.768 ~ -0.518)	< 0.001	0.208^c^
	HPAQ-Self-efficacy	0.773	(0.622 ~ 0.923)	< 0.001	0.208^c^
	HPAQ-Social support	0.671	(0.557 ~ 0.785)	< 0.001	0.257^c^
	HPAQ-Activity-related affect	1.074	(0.879 ~ 1.268)	< 0.001	0.234^c^
Social	Social function total scores	1.874	(1.662 ~ 2.085)	< 0.001	0.440^c^
	-Social adaption	1.454	(0.821 ~ 2.087)	< 0.001	0.050^c^
	-Social support	4.024	(3.405 ~ 4.643)	< 0.001	0.297^c^
	-Social engagement	3.089	(2.703 ~ 0.475)	< 0.001	0.391^c^
**Health self-management (HSM) ability total scores**		0.748	(0.674 ~ 0.823)	< 0.001	0.502^c^
	-HSM-Consciousness	1.518	(1.300 ~ 1.736)	< 0.001	0.326^c^
	-HSM-Environment	1.551	(1.312 ~ 1.790)	< 0.001	0.296^c^
	-HSM-Behavior	1.156	(0.983 ~ 1.330)	< 0.001	0.307^c^
**Self-perceived healthy aging (SPHA)**		12.011	(9.329 ~ 14.693)	< 0.001	0.167^c^

Notes: ^c^Simple linear regression was used; b = for Mean (SD) and c = for r^2^; ^a^Independent t-test; Abbreviations: SRH = Self-rated health (Subjective health); HPAQ = Health promotion activity questionnaires; M = Mean, SD = standard deviations

### Environmental factors

Simple linear regression was used for the analysis between environmental-related data and HA scores as presented in Table 5. FAS (both total and subscale scores), community-based HPAQ (all subscales), the frequency of elderly participation in health promotion activities, and social functions (both total and subscale scores) were significantly associated with healthy aging scores (*p* < 0.05).

### Health self-management ability factors

Insert Table 5 shows that the HSM ability (both total and subscale scores) of the participants was significantly associated with healthy aging scores (*p* < 0.05).

### Self-perceived healthy aging factors

Self-perceived healthy aging (SPHA) scores of the participants were significantly associated with their healthy aging scores (*p* < 0.05) (Table 5).

### Stepwise multivariate linear regression analysis of healthy aging

The stepwise multivariate linear regression model revealed significant predictors of healthy aging, including self-perceived economic independence, drinking alcohol, SRH, depression, BPNS-competence, frequency of elderly participation in health promotion activities, HPA-perceived barriers, social support, social engagement, HSM-Consciousness and HSM-Behavior, and SPHA (Table 6).

**Table 6:** Stepwise multivariate linear regression analysis of predictors of healthy aging scores among older adults in Lishui, China (*n* = 389)

Influential factor	B	SE	β	t value	*p*-value	95% (CI)
(constant)	24.488	14.626		1.674	0.095	(-4.280 ~ 53.256)
Self-perceived Economic independence (Independency = 0, dependency = 1)	-3.640	1.439	-0.086	-2.529	0.012	(-6.470 ~ -0.809)
Drink alcohol	-3.385	1.293	-0.081	-2.617	0.009	(-5.929 ~ -0.841)
Self-rated health	3.059	0.790	0.117	0.141	<0.001	(1.506 ~ 4.612)
Depression	-0.462	0.210	-0.075	-2.202	0.028	(-0.874 ~ -0.049)
BPNS-competence	1.445	0.445	0.106	3.246	0.001	(0.570 ~ 2.321)
Frequency of elderly participation in community-based HPA	1.539	0.579	0.104	2.658	0.008	(0.400 ~ 2.678)
HPAQ-perceived barriers	-0.174	0.052	-0.124	-3.380	< 0.001	(-0.276 ~ -0.073)
SF-social support	0.637	0.309	0.086	2.058	0.040	(0.028 ~ 1.245)
SF-social engagement	0.471	0.233	0.096	2.022	0.044	(0.013 ~ 0.930)
HSM-Consciousness	0.475	0.112	0.179	4.2228	< 0.001	(0.254 ~ 0.696)
HSM-Behavior	0.257	0.086	0.132	3.193	0.002	(0.106 ~ 0.445)
SPHA	2.176	1.014	0.074	2.147	0.033	(0.128 ~ 4.170)

F = 22.744, *p* < 0.001, *R* = 0.854, R^2^ = 0.729, after adjustment R^2^ = 0.697, ^*^P < 0.05, ^**^P < 0.01, ^***^P < 0.001; Abbreviations: BPNS = Basic psychological needs satisfaction; HPAQ = Health promotion activity questionnaires; HSM = Health self-management, SF = Social function, SPHA = Self-perceived healthy aging; B = partial regression coefficient, SE = standard error, β = standard regression coefficient, 95%CI = 95% Confidence interval for B (Lower bound- Upper Bound)

## Discussion

This study reported the prevalence of healthy aging and its associated factors among community-dwelling older adults in Lishui City, a mountainous region in China. The mean score of healthy aging was 136.5 (SD 18.22), much higher than the elderly population in Beijing, the capital city [18]. Out of all the dimensions of healthy aging, seven dimensions recorded higher mean scores while the dimensions of physical activity and cognitive activity had lower mean scores when compared with older adults in Beijing [18]. The highest mean score among the nine dimensions was the dimension of aging acceptance while the lowest score was recorded in the social participation dimension. Overall, the results indicated that older adults from rural mountainous areas had a better acceptance of aging. However, they were less adept at staying physically and cognitively active, as well as less participating socially compared to older adults from urban areas. The findings suggest that the differences in healthy aging among community-dwelling older adults residing in mountainous regions compared to those in urban areas may comprehensively reflect their living conditions in late life. These results provide evidence for a comprehensive understanding of existing problems at specific life stages of the older population, particularly for older adults living in mountainous regions.

In this study, the prevalence of healthy aging among the older population was 65.5%, higher than the prevalence reported in previous studies (3.3-53.15%) [15, 16]. However, the disparity can be attributed to different tools used in measuring healthy aging. The high healthy aging could be the result of the national policies issued by the government to improve the efficiency of the healthcare system, for instance, increasing the supply of low-cost and wide-ranging care for older persons. Similarly, in Zhejiang province, the local policy framework revolves around the improvement of access to rural social care in the community setting [37]. As high as 97% of older adults had at least one type of medical insurance coverage in this study. This reflects the success of the government policies in promoting common prosperity to improve the socio-economic capacity of rural communities. Mountainous areas in Zhejiang province, such as Lishui, were among the first pilot sites where the provincial government implemented strategies to achieve common prosperity [38]. In addition, this study initially provided reference information for both subjective and objective assessments of healthy aging among community-dwelling older adults from mountainous regions under the background of the common prosperity agenda proposed. The subjective perception of good healthy aging (79.7%) among the study population was higher than the prevalence measured by the HAI (65.5%). In other words, the level of healthy aging was high from both objective and subjective perspectives. Because of the successful outcomes in improving healthy aging, similar to the common prosperity programs should be expanded to more areas to reduce urban-rural disparities and health inequalities.

Due to the multidimensionality and complexity of healthy aging, it is challenging to determine the significant associating factors of healthy aging among the diverse range of potential factors that are present in the aging population [39]. This study contributed important data on healthy aging and its association with individual, environmental, and individual-environment interaction factors among older populations from mountainous areas. We identified 12 predictors of healthy aging under this similar component; individual, environmental, individual-environment interaction factors.

This study found economic independence led to high healthy aging score. This finding is consistent with previous studies from Europe [40], Korea [41] and Canada [42]. Economic independence is seen as one of the important foundations enabling proximal factors such as health lifestyle habits, social engagement, effective and accessible access to healthcare, and maintaining independence at home [42], all of which can improve healthy aging among older adults. In contrast, a lack of economic independence represents a barrier for them to access health and social service needs [43], subsequently leading to poor healthcare access and health outcomes [44] and lower healthy aging scores. In China, there is a wide disparity in the level of economic independence between east-west and urban-rural regions. In this study, the economic independence among older adults in Zhejiang province in the east of China is generally higher than their counterparts from rural areas in the west of China [45]. Additionally, policy differences and market reforms among different regions also affect living conditions, social support, socioeconomic status, and health outcomes of the population, all of which can influence the healthy aging scores as reported in previous studies [46–48].

This study found alcohol consumption led to lower healthy aging scores. This finding contradicts a Mexican study whereby older adults who drink alcohol reported higher levels of healthy aging [49]. Previous studies demonstrated that nationality is one of the significant factors associated with alcohol intake [45, 50]. Our study was consistent with several studies that revealed a history of no [51] or reduced intake of alcohol [52] was associated with a low risk of overall mortality and CVD risk in older adults regardless of socioeconomic status. More studies are warranted to explore the impact of cultural influence on the relationship between alcohol intake and healthy aging.

Furthermore, with regard to individual physical health factors, SRH is a more sensitive indicator of the association between older adults’ health condition and healthy aging scores as compared to the association between medical diagnoses (NCDs) or measures of physical functioning alone (number of falls in the past year) with healthy aging scores. We postulated the reason as SRH being a more inherently holistic measure of overall health [53], thus a better screening tool to evaluate older adults’ health status. It also has a solid biological basis and robust association with mortality [54] because it can reflect an individual’s perception and evaluation of preexisting illnesses apart from screening for any undiagnosed symptoms of the illness [55]. As for mental health, depression was associated with healthy aging in this study, in line with previous studies [56, 57]. Depression was associated with a 12% greater hazard risk of losing healthy aging status (chronic disease, non-normal physical functioning, or dying) after 14 years of follow-up in a related study [58]. These results highlight the need for close monitoring and early intervention of depression among older adults, especially those living in mountainous areas. Improved mental health outcomes can enhance their healthy aging. Additionally, the competence domain in the BPNS was found to be directly associated with healthy aging, consistent with self-determination theory [59] and Maslow’s hierarchy of needs [60]. Competence refers to the basic needs of individuals to be able to master new skills and feel efficient [30]. According to Maslow’s hierarchy of needs, competence is a basic individual need throughout the life course and it is located at the middle-high level of the hierarchy pyramid. The basic psychological needs of older adults can help them to operate effectively within crucial life contexts, whereby an individual with a lower BPNS score is associated with poorer physical health and function [61], and worse psychological health such as feeling depressed and useless [62], and decline in social interactions [63], all of which might lead to decreased healthy aging. Therefore, government agencies, health care workers, community members, geriatricians, and family members should pay more attention to enhancing older adults’ BPNS, especially since better competence is expected to improve older adults’ healthy aging.

In addition, the frequency of participation in community-based health promotion activity (HPA) also cast a positive associated with healthy aging, consistent with previous studies [64, 65]. Regular health promotion activities ranging from low-intensity walking to more vigorous sports and resistance exercises should be encouraged among the older population as these activities are safe and can them mobile and independent, subsequently improving their physical and mental functions [64]. A systematic review found that the frequency of elderly participation in health promotion activities plays a crucial role in enhancing healthy aging [65].

As for HPA, the dimension of perceived barriers was associated with healthy aging, similar to previous studies [66]. In addition, previous studies demonstrated that training based on a health-enhancement approach can effectively promote physical activity among older adults [67] and lower their perceived barriers to participating in physical activity [68, 69]. The main reason might be that HPA-perceived barriers can be related to individual, environmental, or the interaction of both factors. In this study, HPA-perceived barriers included individual factors such as physical (aliments, body pain, feeling powerless and weak, aggravated symptoms of existing diseases) and mental (lack of motivation), environmental (far away or no suitable community health activity centers or groups, lack of professional guide or peer support). Apart from that, individual-environmental interactions such as high cost, lack of time, self-perceived aging, and insufficient information also reduced HPA-perceived benefits among older adults and their self-efficacy, thus limiting their social support. In a vicious cycle manner, these barriers may further hinder their utilization of fitness facilities and participation in community activities, reducing their ability to maintain a healthy lifestyle and decreasing their healthy aging.

Next, the two dimensions of social functions (social support and social engagement) also associated with healthy aging. WHO has demonstrated social determinants of health (SDH) as an important element in the promotion of healthy aging [2, 70]. Together with social support from family, friends, and neighbors, an age-friendly environment enables the cultivation of positive social relationships and connectivity [71], hence leading to enhanced social function and improved healthy aging among older adults. Moreover, social engagement is a critical proximal factor of healthy aging [42] as a socially viable lifestyle has been shown to older adults’ subjective health [72] and slow down the decline of cognitive function [73]. In addition, social engagement such as community activities or volunteering activities also heightens older adults’ satisfaction, sense of achievement and value as reported in a previous study [74]. In other words, older adults with better social engagement are generally associated with enhanced healthy aging. However, in this study, the dimensions of social adaptation did not show any significant relationship with healthy aging, in contrast to previous studies [75, 76]. As compared to social support and social engagement, social adaptation is a more fluid concept due to underlying geographical, population, political, and cultural differences. About half of our study participants were farmers (46.8%) with low education levels (53.5%) who likely did not experience much improvement in terms of work and living environment even with increasing age. In an American study, successful societal adaptation to the aging population hinged on adjusting and adapting the relevant practices in all education, work and retirement, as well as health care institutions [77]. Thus, this study provided additional evidence of the need to strengthen various social functions such as social engagement and social support in promoting healthy aging among older adults.

Lastly, the two dimensions of HSM ability (consciousness and behavior) also cast a significant associated with healthy aging, which was supported by the KAP model in previous studies [78]. Older adults’ HSM ability and healthy aging are both lifelong processes that share similar characteristics influenced by a diverse range of physical, social, cultural, and psychological factors [22]. Their self-care ability [79] is associated with health status (loneliness, cognitive status, and risk of frailty) [23] which eventually affects their quality of life and various aspects of their well-being [80]. Based on this study, the KAP model in health promotion can adopt HSM ability as part of the theoretical support in the examination of the complex associations between individual, environment, and both individual-environment interaction factors and healthy aging.

Similar to previous studies, SPHA also showed a significant association with healthy aging [80, 81] as it can mirror older adults’ subjective perspective of healthy aging. A systematic review suggested that SPHA should be used to provide a more comprehensive evaluation of older people’s healthy aging [20]. Previous studies also showed that positive self-perceptions of aging influence the effect of healthy aging programs [81] and their association with various health-related outcomes [80]. To a certain degree, self-perceived healthy aging is closely related to the overall health and well-being of older adults. Therefore, the promotion of positive self-perceptions of healthy aging through interventions and education can be beneficial for the overall health of older adults.

### Limitations

Several limitations must be considered when interpreting the results of this study. Firstly, the cross-sectional design could not verify the temporal sequence of the study variables and the causal links between them. The use of self-reporting questionnaires might also introduce certain biases such as social desirability. Therefore, longitudinal and experimental research is needed to better understand the predictors of healthy aging. Secondly, the results might not be generalized to a non-community-dwelling older adult population due to the specific target population. Additionally, other potential confounding factors warrant consideration. For example, the dependent variable, healthy aging is assessed through the HAI, which encompasses elements such as staying physically active, staying cognitively active, and having social support. Concurrently, the independent variable comprises physical and cognitive health, as well as social participation. The overlap between the factors measured by both the independent and dependent variables suggests a correlation, which may compromise the robustness of the study’s findings. This interrelatedness could impede the interpretation of the results due to the shared components being measured. Furthermore, other factors and variables not considered in the current study might affect the outcomes due to the interconnectedness of many associated factors such as culture and so on. Thus, the omission of certain factors might have led to biased estimates, voiding causal conclusions.

Despite these limitations, there are some strengths to this study. To the best of our knowledge, this is the first study to provide both subjective and objective assessment of healthy aging, thus facilitating a comprehensive evaluation of the healthy aging from the perspective of the older population. This introduced fresh insights into the research associated with healthy aging among community-dwelling older adults in mountainous areas in China. In addition, this study outlines a set of factors associated with healthy aging from the different perspectives of sociodemographic, lifestyle, health conditions (e.g., physical health, mental health, cognitive function), environmental (e.g., family adaption, community-based health promotion activity, participation, and social function) that can complement existing theory from previous studies to assist policymakers in finetuning relevant programs for older population in rural areas.

## Conclusion

This study reported a high prevalence of healthy aging among community-dwelling older adults from Lishui, China and the significant predictors of good healthy aging. The results of this study successfully addressed the existing gap in understanding healthy aging both subjectively and objectively as well as providing a comprehensive overview of the influencing factors of healthy aging among community-dwelling older adults in mountainous areas in Lishui, China. People-centered approaches that involve wider individual and environmental interaction are preferred to enhance older adults’ HSM ability and healthy aging. Collectively, these findings can serve as important guidelines in the development of healthy aging promotion interventions by targeting specific sociodemographic and lifestyle factors as well as their interaction with the living environment to ensure a customized community-based health promotion program that can assist community healthcare staff in improving healthy aging among the older population.

## Data Availability

The datasets used and/or analyzed during the current study are available from the corresponding author upon reasonable request.
